# Disruption of KIF3A in patient-derived glioblastoma cells: effects on ciliogenesis, hedgehog sensitivity, and tumorigenesis

**DOI:** 10.18632/oncotarget.6854

**Published:** 2016-01-09

**Authors:** Lan B. Hoang-Minh, Loic P. Deleyrolle, Dorit Siebzehnrubl, George Ugartemendia, Hunter Futch, Benjamin Griffith, Joshua J. Breunig, Gabriel De Leon, Duane A. Mitchell, Susan Semple-Rowland, Brent A. Reynolds, Matthew R. Sarkisian

**Affiliations:** ^1^ Department of Neuroscience, University of Florida College of Medicine, McKnight Brain Institute, Gainesville, Florida, USA; ^2^ Department of Neurosurgery, University of Florida College of Medicine, McKnight Brain Institute, Gainesville, Florida, USA; ^3^ Preston A. Wells, Jr. Center for Brain Tumor Therapy, University of Florida College of Medicine, McKnight Brain Institute, Gainesville, Florida, USA; ^4^ UF Brain Tumor Immunotherapy Program, University of Florida College of Medicine, McKnight Brain Institute, Gainesville, Florida, USA; ^5^ Cedars-Sinai Regenerative Medicine Institute, Cedar-Sinai Medical Center, Los Angeles, California, USA; ^6^ Samuel Oschin Comprehensive Cancer Institute, Cedar-Sinai Medical Center, Los Angeles, California, USA; ^7^ Division of Applied Cell Biology and Physiology, Cedar-Sinai Medical Center, Los Angeles, California, USA; ^8^ Department of Biomedical Sciences, Cedars-Sinai Medical Center, Los Angeles, California, USA; ^9^ Department of Medicine, UCLA Geffen School of Medicine, Los Angeles, California, USA

**Keywords:** kinesin-2, brain tumor, cilium, intraflagellar transport, sonic hedgehog

## Abstract

KIF3A, a component of the kinesin-2 motor, is necessary for the progression of diverse tumor types. This is partly due to its role in regulating ciliogenesis and cell responsiveness to sonic hedgehog (SHH). Notably, primary cilia have been detected in human glioblastoma multiforme (GBM) tumor biopsies and derived cell lines. Here, we asked whether disrupting KIF3A in GBM cells affected ciliogenesis, *in vitro* growth and responsiveness to SHH, or tumorigenic behavior *in vivo*. We used a lentiviral vector to create three patient-derived GBM cell lines expressing a dominant negative, motorless form of Kif3a (dnKif3a). In all unmodified lines, we found that most GBM cells were capable of producing ciliated progeny and that dnKif3a expression in these cells ablated ciliogenesis. Interestingly, unmodified and dnKif3a-expressing cell lines displayed differential sensitivities and pathway activation to SHH and variable tumor-associated survival following mouse xenografts. In one cell line, SHH-induced cell proliferation was prevented *in vitro* by either expressing dnKif3a or inhibiting SMO signaling using cyclopamine, and the survival times of mice implanted with dnKif3a-expressing cells were increased. In a second line, expression of dnKif3a increased the cells' baseline proliferation while, surprisingly, sensitizing them to SHH-induced cell death. The survival times of mice implanted with these dnKif3a-expressing cells were decreased. Finally, expression of dnKif3a in a third cell line had no effect on cell proliferation, SHH sensitivity, or mouse survival times. These findings indicate that KIF3A is essential for GBM cell ciliogenesis, but its role in modulating GBM cell behavior is highly variable.

## INTRODUCTION

Less than 5% of patients diagnosed with GBM survive over five years following diagnosis, despite resection, chemotherapy, and radiation treatment [1]. Extending this survival period is dependent on improving our understanding of the cellular and molecular pathways that enable GBM cells to survive and thrive within the tumor environment [2, 3]. Multiple studies have reported that the growth of some GBM tumors and glioma-derived stem cells is activated by the SHH signaling pathway [4–9]. A subset of GBM tumors display enhanced SHH signaling, and this can be recapitulated *in vitro*, where some GBM cell lines proliferate in response to SHH treatment [9]. In some tumor types, SHH-driven tumorigenesis is sensitive to the disruption of KIF3A, a subunit of the kinesin-2 motor whose anterograde function is required for ciliogenesis [10–12]. In particular, one of the first reports that studied the function of *Kif3a* in mouse medulloblastoma showed that constitutively active SMO-driven tumor formation is inhibited by loss of KIF3a [13]. A more recent study confirmed this finding, showing that KIF3a is necessary for medulloblastoma initiation and maintenance and that conditional ablation of *Kif3a* levels during tumor formation *in vivo* is followed by tumor regression [14]. A similar observation was reported in basal cell carcinoma in mice, whereby conditional ablation of *Kif3a* blocked hedgehog-driven tumorigenesis [15]. Though not SHH driven, silencing of KIF3a expression in advanced prostate cancer was also reported to suppress cell proliferation and invasion [16]. Despite its observed roles in the previous tumor types, little is known about the roles of KIF3A in GBM.

KIF3A is required for ciliogenesis in certain cell types, and canonical SHH signaling is known to be mediated by the primary cilium (for review see: [17]). SHH binds to its ciliary membrane receptor, Patched, which induces an influx of smoothened (SMO) and Gli transcription factors into the cilium. These proteins trigger the activation of other downstream Gli transcription factors that can, among other effects, increase mitogenesis [18–20]. Despite the known continued synthesis of SHH in the adult brain and by some GBM cells [4, 21, 22], it remains unclear whether ciliary SHH signaling contributes to GBM tumor growth. The reported percentages of cells that possess primary cilia in tumor biopsies and in different GBM cell lines are quite variable [23, 24]. For instance, less than 1-2% of the widely studied astrocytoma and GBM cell lines (U-87MG, T98G, U-373MG, U-251MG) have been reported to assemble fully formed primary cilia in some studies [23]. In our recent analyses of 23 GBM patient biopsies and 5 primary derived cell lines, we identified well-formed primary cilia on ∼8-25% of the GBM cells examined at any given point in time [24]. The functional significance of the cilia associated with these subpopulations of GBM cells has not yet been determined. A previous study reported that knockdown of Kif3a in U251-MG cells by siRNA slightly reduced the percentage of ciliated cells (from 2% to 1%), but did not have an appreciable effect on cell proliferation or cell cycle phase distribution *in vitro* [25]. Thus, we wondered whether our patient-derived GBM cell lines, which display a significantly higher frequency of cilia than the commonly studied U-lines, would be more sensitive to the disruption of KIF3A.

The purpose of this study was to first disrupt KIF3A in primary GBM cell lines through lentiviral expression of dnKif3a [26, 27] and characterize the resulting effects on ciliogenesis. We also determined whether these modified cell lines showed altered proliferation and/or sensitivity to SHH *in vitro*, and whether disrupting KIF3A function affected mouse survival following xenotransplantation.

## RESULTS

### The majority of patient-derived GBM cells give rise to ciliated progeny

**Figure 1:**
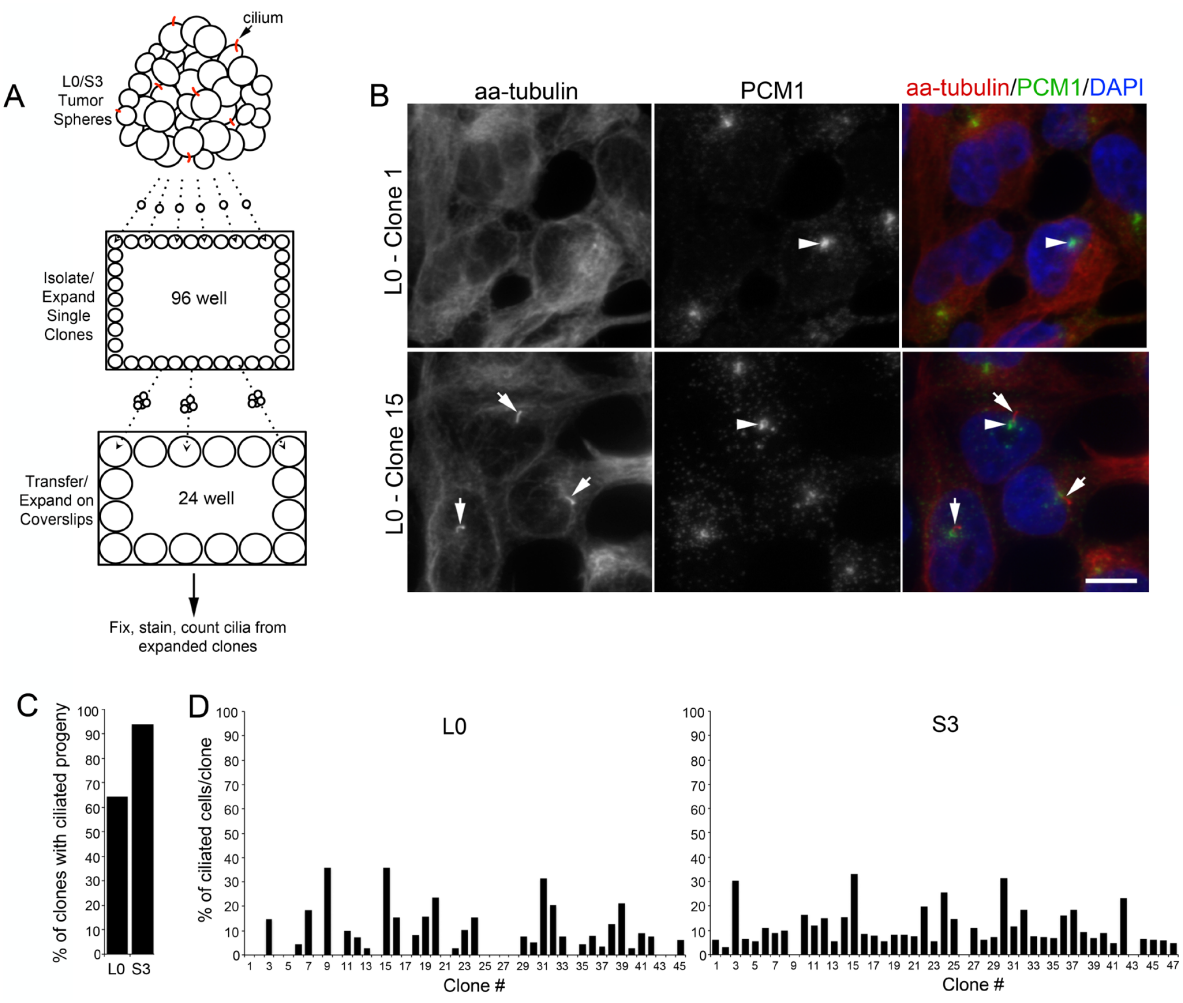
A majority of isolated patient-derived GBM cells produce ciliated progeny (**A**) Spheres of Line 0 (L0) and S3 GBM cells were dissociated, sorted as single cells into 96-well plates, and further expanded onto coverslips in 24-well plates prior to immunostaining for cilia markers after 24 hours. (**B**) Confocal maximum projection images of L0 clones (1 and 15) immunostained for PCM1 (green) and acetylated alpha-tubulin (aa-tubulin; red). The merged images (right panels) show aa-tubulin-positive axonemes (arrows) projecting from PCM1-positive basal bodies (arrowheads) in Clone 15 but not in Clone 1. (**C**) Percentage of all clones in L0 or S3 cell lines that gave rise to ciliated progeny. (**D**) Percentage of L0 or S3 cells with aa-tubulin-positive cilia derived from individual clones. Scale bar in B = 10 μm.

The formation of cilia depends on the kinesin-2 motor, which includes KIF3A, in various cell types (for review see: [10, 28]). It is not clear what percentage of GBM cells have the capability to form cilia. We previously reported that ∼8-25% of the cells from GBM cell lines which we derived from patient tumors were ciliated at any given point in time in our cultures [24]. Here, we asked whether those percentages accurately reflect the fraction of cells capable of generating cilia, that is, are only 8-25% of GBM cells capable of ciliogenesis, or can more cells give rise to ciliated offspring? To answer this question, spheres of L0 and S3 GBM cells were dissociated, plated as single clones, and expanded. We then quantified the number of clone-derived populations containing ciliated cells (Fig. 1A). Cilia were visualized using antibodies recognizing acetylated alpha-tubulin (aa-tubulin) and PCM1, both of which are enriched in the axoneme and in the region surrounding the basal bodies of most cilia, respectively (Fig 1B) [24, 29–31]. We found that 64.4% (29/45) of L0 clones and 93.6% (44/47) of S3 clones gave rise to ciliated cells (Fig. 1C), the numbers of which varied between clone-derived populations (Fig. 1D). Thus, these results suggest that the majority of L0 and S3 GBM cells are capable of generating ciliated progeny.

### Expression of dnKif3a ablates ciliogenesis in three primary GBM cell lines

**Figure 2:**
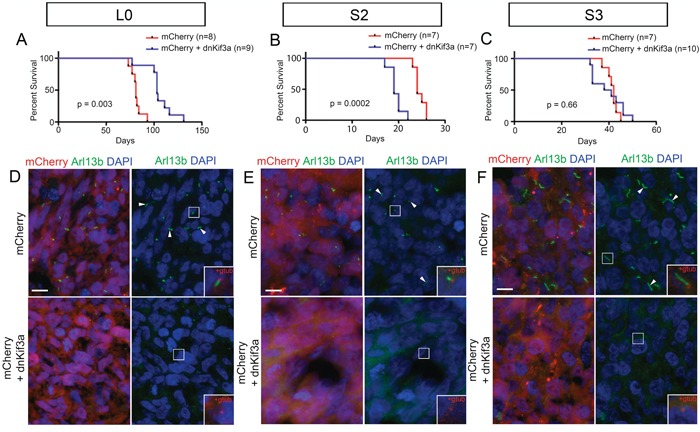
The expression of dnKif3a potently inhibits cilia formation in three GBM cell lines L0, S2 and S3 cell lines were infected with lentiviral vectors encoding either mCherry alone or mCherry and dominant negative Kif3a (dnKif3a) and their cilia examined using immunocytochemistry and EM. Next, we used FAC-sorting to select mCherry-positive L0, S2, and S3 cells, expanded the sorted cells, and assessed whether they were able to form cilia using ultrastructural and immunocytochemical techniques. (**A**) Confocal image shows L0 cells (prior to sorting) with aa-tubulin-positive cilia on infected mCherry-positive cells (arrows) and non-infected mCherry-negative cells (arrowhead). (**A'**) Sorted mCherry-positive cells were fixed and analyzed by EM. The example shows a cilium with a docked basal body (arrow) and a long ciliary axoneme (arrowheads) projecting outside the cell. (**B**) L0 cells (prior to sorting) revealed aa-tubulin-positive cilia but only on non-infected (mCherry-negative) cells (arrowheads) and not on infected mCherry and dnKif3a-positive cells. (**B', B”**) By EM, sorted L0 mCherry and dnKif3a-positive cells revealed basal bodies (arrows) that lacked a clear axoneme (**B'**; arrowhead) or displayed an abnormally assembled/poorly formed axoneme (**B”**; arrowhead). (**C**) Percentage (+/− SEM) of aa-tubulin-positive ciliated cells among sorted mCherry-positive and sorted mCherry and dnKif3a-positive cells for the indicated cell lines. (**D**) Endogenous KIF3A levels are reduced in L0, S2 and S3 GBM cell lysates after dnKif3a expression. Thirty μg of total protein lysates from L0, S2 and S3 cells expressing mCherry or mCherry and dnKif3a were separated by SDS-PAGE and western blotted with an antibody against human KIF3A protein. Although KIF3A (∼ 85 kDa) was detected in all groups, lysates from mCherry and dnKif3a-expressing cells consistently displayed reduced KIF3A levels compared to those expressing mCherry alone. We also observed a smaller band (∼ 40 kDa, arrowhead) in lanes with dnKif3a-expressing cell lysates, which might either represent degraded endogenous KIF3A or possibly a sequence associated with the expression of dnKif3a. Δ-actin was run as a loading control. Scale bars for A and B = 10 μm; A' = 500 nm; B' and B” = 250 nm. ***p<0.005 (Student's t-test).

Because the majority of GBM cells that we examined gave rise to ciliated cells, we next determined whether the formation of these cilia depended on KIF3A. In previous studies, our group and others showed that cilia formation could be blocked by interfering with the function of Kif3a through the expression of its dominant negative form (dnKif3a), a motorless form of the molecule that inhibits ciliogenesis [26, 27]. We infected three of our GBM cell lines that are able to elaborate cilia (L0, S2, and S3) with lentiviral constructs expressing mCherry or mCherry and dnKif3a. About ten days after transduction, cilia immunostained for aa-tubulin were easily detected on both mCherry-positive and non-infected cells (Fig. 2A), but were not observed on mCherry-positive cells expressing dnKif3a (Fig. 2B). Using FACS, we selected mCherry-positive L0, S2, and S3 cells that had been infected with either of these viruses, expanded the selected cells, and examined their ability to form cilia using both ultrastructural and immunocytochemical techniques. Using electron microscopy (EM), we found that the mCherry-positive L0 control cells extended well-formed cilia (Fig. 2A'). In contrast, mCherry-positive L0 cells that had been infected with the mCherry and dnKif3a-expressing lentiviral construct either failed to elaborate cilia (Fig. 2B') or developed cilia with aberrantly formed axonemes arising from their basal bodies (Fig. 2B”). These observations with EM confirmed our immunocytochemical analyses. Quantification of immunostained infected cells revealed that 29±4.9%, 11±2.3%, and 16±1.1% of the L0, S2, and S3 mCherry-positive cells expressing the control transgene were ciliated, respectively (Fig. 2C). However, very few (≤ 1%) of the sorted mCherry-positive cells expressing dnKif3a elaborated cilia (Fig. 2C), which, if present, were similar to the short buds observed in our EM analyses of these cells (Fig. 2B”). Thus, the expression of dnKif3a disrupted the ability of L0, S2, and S3 GBM cells to assemble cilia. It is also noteworthy that the inability to synthesize cilia persisted across cell passages, indicating that integration of the transgenes were stable in these modified cell lines.

Next, we examined the effects of persistent expression of mCherry alone or mCherry and dnKif3a on endogenous human KIF3A levels in GBM cells, as our dnKif3a transgene was derived from mouse *Kif3a* [27]. Based on our results above, we expected that the human KIF3A levels would have been altered, since the expression of the mouse dnKif3a protein disrupted ciliogenesis. Western blots were prepared using protein lysates extracted from each sorted cell line and were probed with an antibody to KIF3A. We found that the levels of human KIF3A in L0, S2, and S3 cells expressing dnKif3a were consistently lower than those detected in control cells (Fig. 2D). Thus, the disruption of ciliogenesis could arise from either outcompetition of endogenous KIF3A by dnKif3a or reduced levels of human KIF3A in our GBM cells expressing mCherry and dnKif3a. At this point, we do not know the exact mechanism that is responsible for the disruption of ciliogenesis in our dnKif3a-expressing cell lines; however, whatever the mechanism, our results are consistent with practically every other study in which targeting KIF3A function and/or expression levels interferes with cilia formation [14, 15, 19, 20, 26, 27, 32].

### Disruption of KIF3A has cell line-specific effects on SHH sensitivity and GBM cell proliferation

**Figure 3:**
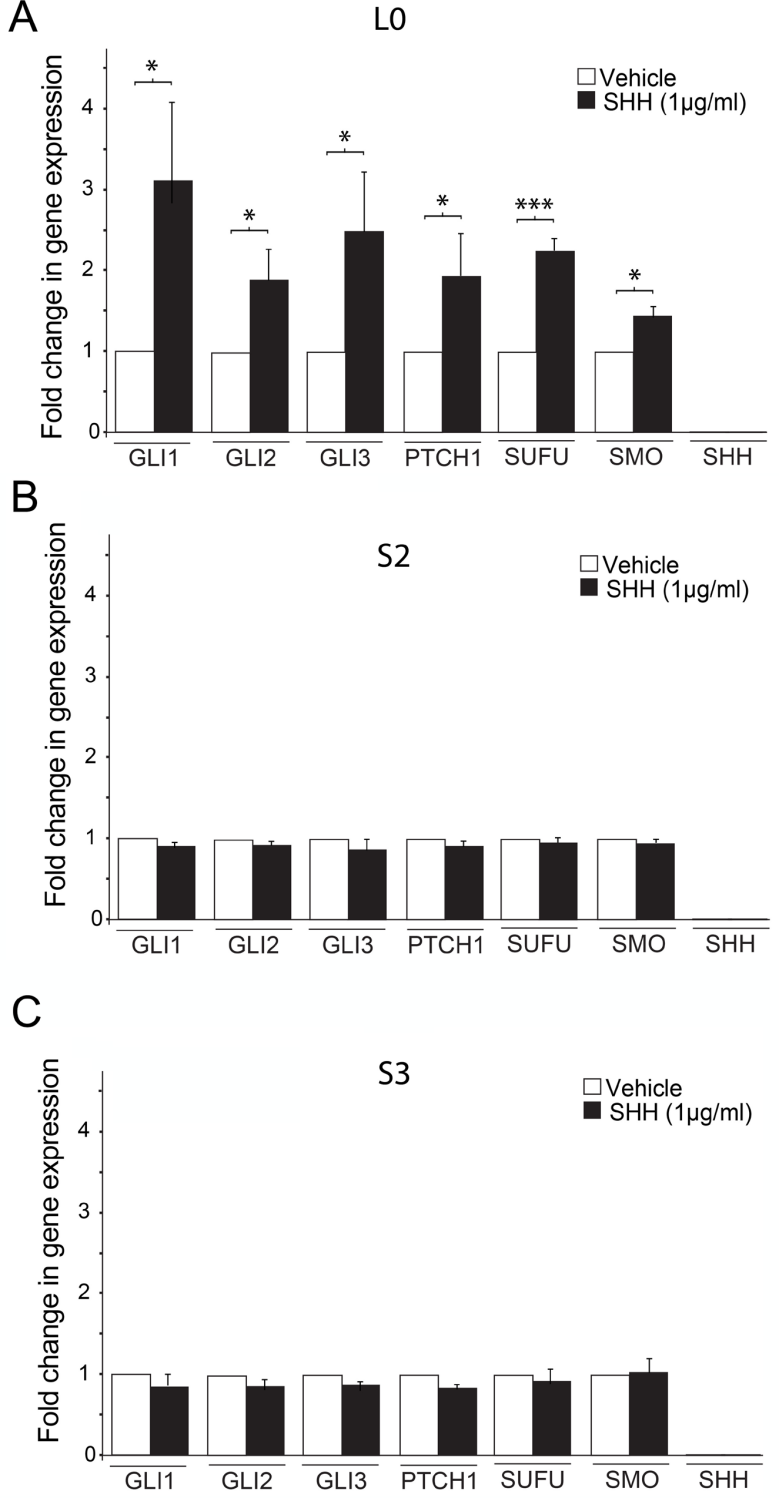
The effects of dnKif3a expression and SHH on GBM cell proliferation are cell-line dependent Mean cell numbers (+/− SEM) per well for L0 (**A**), S2 (**B**) and S3 (**C**) mCherry-positive and mCherry and dnKif3a-positive cells five days after exposure to vehicle or recombinant human SHH (1 μg/ml), with or without prior addition of cyclopamine (1 μM). Groups were compared using a one-way ANOVA followed by Tukey's posthoc analysis. *p<0.05, ***p<0.005.

Since we had found that KIF3A was critical for GBM cilia formation, and other studies had shown that KIF3A was essential for cilia-mediated SHH signaling in some tumors and SHH pathways are active in GBM, we next examined whether loss of cilia in dnKif3a-expressing cells would change these cells' responsiveness to SHH. First, to examine the sensitivity of our GBM cell lines to SHH, we exposed dissociated control and dnKif3a-expressing/cilia-ablated L0, S2, and S3 cells to vehicle or recombinant human SHH (1 μg/ml) in growth media in the absence of bFGF, EGF, and serum for five days and assessed the levels of cell proliferation. Analyses of the effects of SHH on the proliferation of the L0 and L0-dnKif3a cell lines revealed that SHH treatment significantly increased (p < 0.001; one-way ANOVA) the proliferation of the L0 cells *in vitro*, which was blocked by pretreatment of the cells with cyclopamine (1 μmol/l), a drug that binds to and blocks SMO entry into the cilium [33]. On the other hand, SHH treatment did not affect the proliferation of the L0-dnKif3a cells (Fig. 3A). Pretreatment of the L0 cells with cyclopamine did not alter the proliferation of L0 cells treated with vehicle alone, a result showing that the increase in proliferation observed in response to SHH treatment was due to the addition of SHH and not an autocrine pathway (Fig. 3A). As canonical SHH signaling is known to be mediated by the primary cilium, leading to increased mitogenesis in certain cell types (for review see: [17]), we examined the ability of cilia on L0 cells to recruit components of the SHH signaling pathway. We found that both endogenous SMO and endogenous GLI3 were recruited to the cilia of L0 cells 24 hours following SHH treatment (Supplemental Fig. S1), observations consistent with previous reports of SHH signaling pathway activation [33–35]. Together, these data strongly suggest that L0 cells engage their cilia in response to SHH, stimulating the proliferation of these cells.

In contrast, SHH treatment of S2 control cells did not stimulate their proliferation (Fig. 3B). On the other hand, we found that pretreatment of these control cells with cyclopamine reduced total cell numbers when cells were subsequently treated with SHH (Fig. 3B). Total S2-dnKif3a cell numbers were also reduced when these cells were treated with SHH (Fig. 3B). Interestingly, exposure of S2-dnKif3a cells to SHH or cyclopamine reduced cell viability and, in the case of SHH, increased apoptotic cell death as assessed by MTT, Trypan blue exclusion, and activated caspase-3 staining assays, respectively (Supplemental Fig. S2). Thus, SHH exposure following blockade of ciliary SMO with cyclopamine or expression of dnKif3a/ablation of cilia decreased overall S2 cell proliferation. Intriguingly, we found that dnKif3a expression/cilia ablation in S2 cells correlated with a significant increase in their baseline proliferation, in the absence of SHH treatment (Fig. 3B). Thus, for S2 cells, our data suggest that KIF3A, and possibly primary cilia, play a role in restraining cell proliferation.

Finally, analyses of the S3 cell line revealed that neither SHH treatment, cyclopamine pretreatment, nor dnKif3a expression had any appreciable effects on cell proliferation (Fig. 3C). These results suggest that S3 cells are either insensitive to SHH stimulation, or that their ability to respond to SHH through their cilia is defective, possibilities that would warrant further investigations. Taken together, our data suggest that the presence of KIF3A, and possibly primary cilia, has heterogeneous effects on the proliferation and survival of GBM cells.

### SHH signaling pathway gene expression in the absence or presence of SHH is cell line-dependent

**Figure 4:**
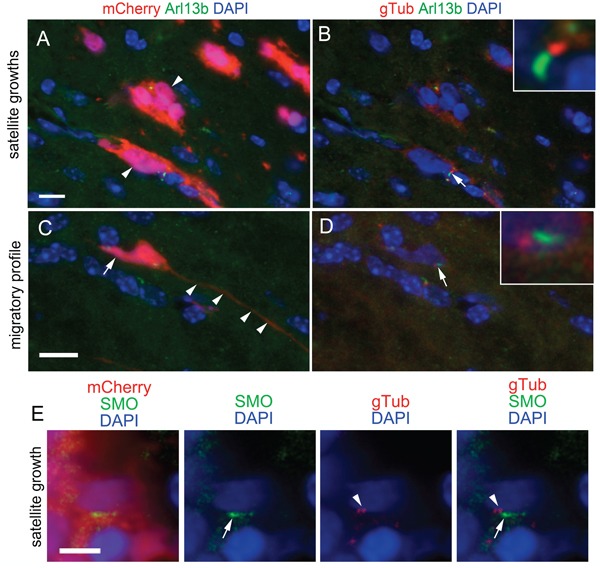
SHH treatment induces changes in SHH signaling pathway gene expression in L0, but not S2 and S3 cell lines (**A-C**) The mRNA expression levels of *GLI1*, *GLI2*, *GLI3*, *PTCH*, *SMO*, *SHH*, and *SUFU* were estimated by real-time qPCR. Increases in SHH pathway gene expression were observed in the L0 (**A**) but not S2 (**B**) or S3 (**C**) cell line (n = 3 biological replicates/gene). The expression of each target gene was quantified relative to *β-actin* mRNA. Data are expressed as means ± SEM. *p<0.05, **p<0.01, ***p<0.005 (Student's t-test).

The different cell line responses to SHH treatment in our cultures prompted us to directly measure whether SHH pathway components' level and activity were different between cell lines. We assessed the basal and SHH-induced expression levels of seven major SHH pathway members (*GLI1*, *GLI2*, *GLI3*, *PTCH*, *SMO*, *SHH*, *SUFU*), 24 hours after cells were treated with either vehicle or SHH. In the L0 cells, we found significant increases in transcript levels for all SHH pathway components, except *SHH*, after SHH treatment compared to vehicle-treated control (all p < 0.05) (Fig. 4A). For the S2 and S3 cell lines, no changes in SHH pathway components' expression occurred after SHH treatment (Fig. 4B, C), a result that is consistent with the unchanged cell proliferation observed following SHH addition in culture (Fig. 3B, C). These data suggest that the SHH-induced proliferative response in L0 cells might be due to SHH pathway activation in those cells, which doesn't occur in S2 or S3 cells. It is noteworthy that *SHH* was not expressed in L0, S2, or S3 cells before or after SHH treatment, suggesting these cell lines are not using autocrine SHH signaling to proliferate, consistently with our *in vitro* data showing that treatment with cyclopamine alone had no effect on GBM cell proliferation.

### Expression of dnKif3a in transplanted GBM cells affects mouse survival times in a cell line-dependent manner

**Figure 5:**
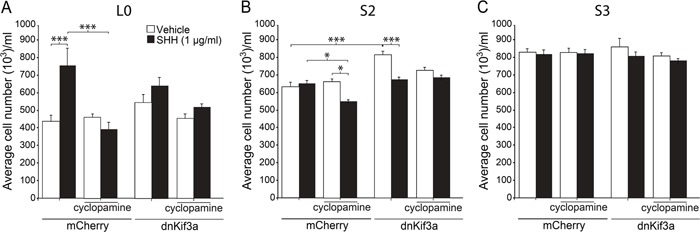
The relationship between dnKif3a expression and mouse survival following intracranial xenograft is cell-line dependent (**A-C**) Kaplan-Meier curves for mice xenografted with mCherry-positive control (red) or mCherry and dnKif3a-positive (blue) L0 (**A**), S2 (**B**) and S3 (**C**) cells. n = number of mice per group. (**D-F**) Sections through the mCherry-positive primary tumor mass immunostained for Arl13b, a marker of primary cilia. In L0 (**D**), S2 (**E**) and S3 (**F**) cell lines, we detected numerous Arl13b-positive cilia (arrowheads) in mCherry-positive control tumors, but not in tumors comprised of cells expressing mCherry and dnKif3a. Boxed regions in D-F are enlarged in the right panel insets to show that, in conjunction with staining for gamma-tubulin (gtub), Arl13b-positive cilia extend from gamma-tubulin-positive basal bodies (red) in mCherry control but not mCherry and dnKif3a-positive cells. Scale bars in D-F = 10 μm.

In this series of experiments, we examined whether expressing dnKif3a, which ablated GBM cell cilia, influenced the ability of GBM cells to form tumors within the brain tumor milieu. The right striata of adult male NOD/SCID mice were injected with 200,000 control or dnKif3a-expressing L0, S2 or S3 cells, all of which were also expressing mCherry. The mice were cared for in the vivarium until they appeared moribund, at which time they were perfused and their brains processed for histological analyses. We found that the average survival time of mice transplanted with L0-dnKif3a cells (n = 9) was significantly longer than that of mice transplanted with control L0 cells (n = 8) (log rank test; p = 0.003; Fig. 5A). In contrast, the average survival time of mice transplanted with S2-dnKif3a cells was significantly shorter than that of mice transplanted with S2 control cells (n = 7 control, 7 dnKif3a; log rank test; p = 0.0002; Fig. 5B). Similar survival times were observed in mice transplanted with either S3-dnKif3a cells or S3 control cells (n = 7 control, 10 dnKif3a; log rank test; p = 0.66) (Fig. 5C).

Histological analyses of the brains of all of the injected mice carried large mCherry-positive tumors (data not shown) that displayed characteristic features of typical GBM tumors such as pseudopalisading necroses and hyperplastic blood vessels (Supplemental Fig. S3). Sections of the cores of each tumor were immunostained for Arl13b, a GTPase that is enriched in the axonemes of most primary cilia [36–38], and gamma (G)-tubulin, a protein that is abundant in basal bodies, to verify that the mCherry-positive L0, S2 and S3 control cells remained ciliated and that the mCherry-positive L0, S2, and S3 cells expressing dnKif3a were not. As expected, numerous mCherry-positive cells in tumors generated from control L0, S2 or S3 cells possessed Arl13b- and G tubulin-positive cilia, whereas those generated from L0, S2, and S3 cells expressing dnKif3a lacked Arl13b- and G tubulin-positive ciliated cells (Fig. 5D-F). We were also unable to detect aa tubulin-positive cilia axonemes in tumors generated from the cell lines expressing dnKif3a, confirming the Arl13b- and G-tubulin staining results (Supplemental Fig. S4).

**Figure 6:**
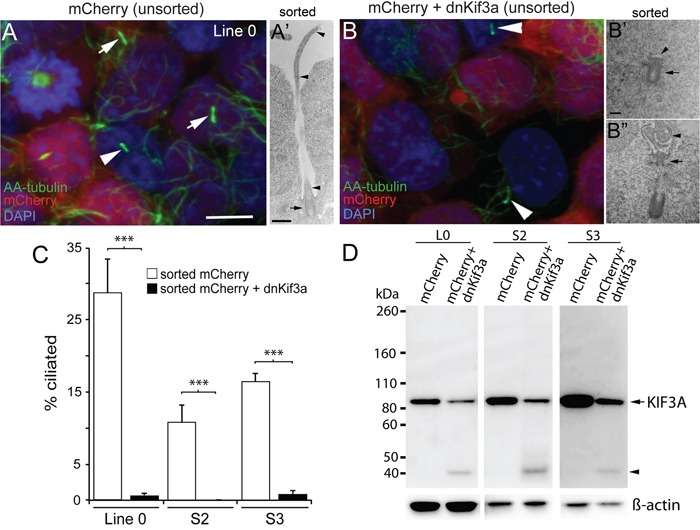
Ciliated, SMO-positive L0 cells are found in sites distal to the tumor mass (**A**) Image of mCherry and Arl13b-positive cells (arrowheads) in satellite growths that formed outside of the primary tumor core in L0 control cell-derived tumors. (**C**) mCherry-positive cell displaying a migratory profile with a short trailing process (arrow) and long leading process (arrowheads). (**B, D**) Gamma-tubulin (gTub) (red) was substituted for mCherry in A and C, respectively. Arrows point to Arl13b-positive cilia with G-tubulin-positive basal bodies enlarged in insets. (**E**) Example of an mCherry-positive cell observed away from the primary tumor mass displaying gamma-tubulin positive basal bodies (arrowheads) and harboring a SMO-positive cilium (arrows). Scale bars for A and C = 10 μm; E = 5 μm.

Our finding that the average survival time of mice transplanted with L0 control ciliated cells was significantly shorter than that of mice transplanted with L0-dnKif3a/cilia-depleted cells, together with the observation that SHH treatment of L0 control ciliated cells increased their proliferation *in vitro*, prompted us to examine whether ciliated cells were capable of migrating beyond the periphery of the primary tumor mass, where they could potentially receive and respond to SHH signals present in the adult brain environment [21, 22]. Indeed, we found ciliated L0 cells that had migrated or appeared to be migrating away from the core and were associated with satellite tumor growths (Fig. 6A-D). Next, we immunostained the brain tumor sections with antibodies against gamma-tubulin, a marker of ciliary basal bodies, and SMO, a key component of the ciliary SHH signaling pathway that is transported into the cilium when the cilium responds to SHH, to determine whether there was any evidence that the cilia of distally located L0 cells had been responding to SHH prior to brain fixation. In rare instances, we found scattered mCherry-positive L0 control cells that were located outside of the primary tumor mass and stained positively for gamma-tubulin and SMO (Fig. 6E), observations suggesting that the cilia of these L0 cells were responding to SHH within the brain environment.

## DISCUSSION

Our results show that targeting KIF3A by lentiviral delivery of dnKif3a in GBM cells is a potent method for disrupting ciliogenesis. However, the effects of expressing dnKif3a on cell proliferation, sensitivity to SHH, and tumor-associated survival in mice are all cell-line specific (summarized in Table 1). Our findings raise the possibility that the progression of GBMs for which SHH stimulates cell proliferation (e.g., L0) may be inhibited by the disruption of KIF3A. Alternatively, our findings with S2 and S3 cells suggest that the rate of GBM progression may be accelerated or unchanged by the disruption of KIF3A.

**Table 1:** Cell line-dependent effects of SHH treatment and/or dnKif3a expression on cell proliferation, SHH sensitivity, cell survival and tumor-associated survival

GBM Cell Line			
Treatment	L0	S2	S3
Addition of SHH	Increased proliferation Increased expression of SHH signaling pathway components	No effect No effect	No effect No effect
Expression of dnKif3a	Cilia ablation Inhibition of SHH-induced proliferation	Cilia ablation Sensitization to SHH-induced death Increased baseline proliferation	Cilia ablation No effect
Inhibition of SMO	Inhibition of SHH-induced proliferation	Sensitization to SHH-induced death	No effect
Intracranial implantation of dnKif3a-expressing cells	Prolonged mouse survival	Shortened mouse survival	No effect

Our studies provide the first account of the high prevalence of GBM cells capable of generating ciliated progeny in patient GBM-derived cell lines. At any given time, only 30% of GBM cells may be observed to harbor cilia, most likely because the rest of the cells are actively dividing and not in the G0/G1 phase, when cilia are typically present. It is also possible that our estimates of the percent of clones capable of forming cilia were lower than the actual percentage since, over time, some of the non-ciliated cell populations could have started to form cilia. Therefore, treatments aimed at affecting ciliary functions in our experiments, such as SHH/cyclopamine and dnKif3a expression, most likely affected most/all of the GBM cells during the ciliated stage of their cell cycle. The ability of dnKif3a expression to ablate these cilia is a useful readout for our successful interference with one function of KIF3A. However, caution must be taken before concluding that the effects of dnKif3a expression on GBM cells are solely attributable to cilia dysfunction, as extra-ciliary roles for KIF3A have also been described [39–42]. It is notable, though, that previous studies have reported that *Kif3a* ablation in fibroblasts does not affect their baseline proliferation, although their response to cilia-mediated SHH agonists is impaired [14]. Thus, it is likely that some of the effects we observed are heavily influenced by cilia depletion following dnKif3a expression, and not by extra-ciliary functions of KIF3A.

The results of our experiments with the L0 cell line may provide the first evidence that the effects of SHH on some GBM cells are mediated by primary cilia. We found that inhibiting ciliogenesis by expressing dnKif3a in these cells, or blocking SMO entry into the cilia by pretreating them with cyclopamine [33], disrupted the ability of SHH to induce these cells' proliferation. Importantly, we found that the survival times of mice that received xenotransplants of L0-dnKif3a cells were significantly prolonged compared to those of mice transplanted with control cells, which displayed evidence of ciliary SHH signaling *in vitro* as well as *in vivo*. The prolonged survival times observed in the mice that received dnKif3a-expressing/cilia-depleted L0 cell implants may partly be due to the reduced responsiveness of these cells to endogenous SHH and/or reduced tumor stem cell proliferation, a possibility supported by studies showing that cyclopamine pre-treatment of certain GBM cell lines ablates the glioma stem cell pool, blocks its self-renewal, and inhibits tumorigenesis [4, 5]. Future studies, however, will need to thoroughly examine whether the prolonged survival of mice carrying dnKif3a-expressing cells is attributable to the ciliary and/or non-ciliary roles of KIF3A during the tumorigenesis process.

In contrast with the L0 cell line, the S2 cell line did not show any increase in cell proliferation in response to SHH. It is noteworthy that, in other studies, only a minority of GBM cell lines were observed to be SHH-sensitive [4, 5, 9, 43] and SHH treatment of other tumors, such as lung adenocarcinoma A549 and lung squamous H520 carcinoma cells [44], also had no effect on cell proliferation. On the contrary, and surprisingly, SHH exposure of dnKif3a-expressing/cilia-depleted S2 cells significantly reduced total and viable cell numbers and increased cell death/apoptosis. SHH exposure of S2 control cells that were pre-treated with cyclopamine also significantly reduced total and viable cell numbers. The mechanisms underlying this phenomenon are unclear and differ from other findings reporting that SHH's interaction with its receptor Patched inhibits apoptotic cell death [45]. In addition, cyclopamine treatment of dnKif3a-expressing S2 cells resulted in decreased viable cell numbers. Interestingly, previous studies have shown that cyclopamine can have off-target effects by inducing apoptosis in human medulloblastoma cells [46]. Thus, our data raise the possibility that ciliary SMO may promote S2 cell proliferation/survival. In contrast, we found that the baseline proliferation of dnKif3a-expressing S2 cells was significantly increased compared to S2 control cells *in vitro*, a response that may have contributed to the decreased survival times observed in the mice xenotransplanted with S2 dnKif3a-expressing cells. These results suggest that, in the absence of KIF3A and/or cilia, these cells may possess increased tumorigenic activity, a phenomenon that has been observed in other cancers. For example, in basal cell carcinoma, loss of *Kif3a* and cilia has been reported to accelerate Gli2-activated tumor growth [15]. The opposite phenomenon has been observed when ciliogenesis is induced. For instance, in U251 GBM cells that mostly lack cilia [23], reducing the expression of cell cycle-related kinase increased the frequency of ciliated cells and slowed the proliferation of these cells *in vitro* [25]. Thus, disrupting KIF3A appears to have a dual effect on S2 cells: on one hand, KIF3A disruption sensitizes the cells to SHH-mediated cell death while, on the other hand, it causes cell proliferation to become less restrained, triggering the cells to expand more rapidly and resulting in decreased animal survival *in vivo*.

The S3 cells did not exhibit any proliferative changes, whether baseline or in response to SHH stimulation, regardless of their ciliated state. There were also no differences in survival times between the mice xenotransplanted with S3-dnKif3a cells and those that received control cell implants. Therefore, compared to L0 and S2 cells, disruption of KIF3A in S3 cells did not noticeably alter the growth patterns of the tumor cells, *in vitro* or *in vivo*.

Surprisingly, despite the expression of a majority of SHH signaling pathway components in S2 and S3 cells, SHH treatment failed to induce an increase in the expression levels of these components. It is possible that this response is significantly delayed in those two cell lines or that there are mutations in key pathway members/receptors, which resulted in the failure to induce any response. It is noteworthy that the commonly studied U-87MG, T98G, U-373MG, U-251MG cell lines do not display GLI activity in response to exogenous SHH [47]. Whether the lack of responsiveness to SHH treatment in these cell lines is due to cilia-dependent or independent processes will require further investigation.

Collectively, our results shed new light on the heterogeneous effects of targeting KIF3A in GBM cells. It is possible that screening patients' GBM cell responses to cilia-associated mitogens such as SHH could help predict which patients may respond to treatment modalities that would interfere with the KIF3A/ciliary pathway.

## MATERIALS AND METHODS

### Cell culture and treatment

Three primary cell lines, Line 0 (L0) (43 yo male), Line SN179 (S2) (50 yo male), and Line SN186 (S3) (75 yo male) that were developed from cells isolated from human patient GBM tumors were used in this study [24, 48–51]. The cells were grown as floating spheres and maintained in DMEM/F12 medium that was supplemented with 2% B27, 1% penicillin-streptomycin, 20 ng/ml human EGF, and 10 ng/ml human bFGF. DMEM/F12 medium, B27, EGF, bFGF, and antibiotics were obtained from Gibco (Life Technologies, CA). All cells were grown in a humidified incubator at 37°C with 5% CO_2_. When the spheres reached approximately 150 μm in diameter, they were enzymatically dissociated with Accumax (Innovative Cell Technologies, Inc.) for 10 min at 37°C. Cells were washed, counted using Trypan blue to exclude dead cells, and re-plated in fresh medium supplemented with hEGF and bFGF. For cells grown on glass coverslips, DMEM/F12 medium was supplemented with 5% fetal bovine serum (FBS).

For single cell clonal analyses, spheres of L0 and S3 cells growing in T25 flasks were enzymatically dissociated (Accumax) and individual cells were seeded into 96-well plates containing 250 μL of DMEM/F12 medium supplemented with hEGF and bFGF using a BD FACS Aria II Cell Sorter (BD Biosciences, San Jose, CA). Cell debris were excluded prior to seeding by forward- and side-scatter gating. When the spheres formed from the singles cells were >100 μm in diameter, they were mechanically dissociated, and cells were reseeded into 24-well plates on glass coverslips in DMEM/F12 medium supplemented with 5% FBS. After 2-3 days, the cells were fixed with 4% paraformaldehyde in 0.1 M phosphate buffer (4% PFA) for immunohistochemical analyses as described below.

For SHH and cyclopamine experiments, spheres of L0, S2, and S3 control and dnKif3a-expressing cells were dissociated and plated at densities of 50,000 cells per ml in 24-well plates in serum-free medium lacking hEGF or bFGF. Twenty-four hours later, cyclopamine (1 μmol/l; EMD Chemicals) was added to each culture, followed by the addition of either recombinant human SHH (1 μg/ml; R&D Systems) dissolved in vehicle (PBS containing 0.1% bovine serum albumin) or vehicle alone 30 min later. The number of cells in each well was counted five days after treatment (12 wells per group/experiment). This experiment was repeated 2-3 times for each cell line.

### Generation and characterization of lentivirus-treated GBM cells

To deliver a transgene encoding dnKif3a to cells, we generated a lentivirus carrying a bicistronic transgene encoding AU1-tagged mCherry and dnKif3a driven by an EF1a promoter. Co-expression of AU1-tagged mCherry and dnKif3a in this transgene was facilitated by insertion of a porcine *Teschovirus* (pTV1) 2A-like cleavage peptide between the two ORFs encoding the proteins [52]. We transduced our ciliated GBM cell lines (L0, S2, and S3) with this lentiviral construct, and control cells were transduced with a lentivirus encoding mCherry (AU1) alone under the control of the same promoter. Two microliters of either lentiviral vector were added to T25 (5 ml flasks) containing L0, S2 and S3 cells immediately following passage (∼50-100 passages from original harvest). Approximately 10 days after transduction, the infected cells were fixed and immunostained for acetylated alpha (aa)-tubulin-positive cilia and analyzed by EM.

### Antibodies

Primary antibodies used for immunocytochemistry (ICC) or immunohistochemistry (IHC) included mouse anti-acetylated alpha-tubulin (1:3000 (ICC/IHC); Sigma (cat # T6793; lot # 088K4829)), rabbit anti-Arl13b (1:3000 (IHC); Proteintech (cat # 17711-1-AP; lot # 00017960)), mouse anti-gamma-tubulin (1:2000 (ICC/IHC); Sigma (cat # T6557; lot # 072M4808)), rabbit anti-Gli3C (1:1000 (ICC); gift from S. Scales) [35], rabbit anti-PCM1 (1:1000 (ICC/IHC); Bethyl Laboratories (cat # A301-150A; lot # A301-150A-1)), rabbit anti-SMO (1:1000 (ICC/IHC); Abcam (cat # ab38686; lot # GR198520)). Cells and sections stained sequentially with mouse antibodies against gamma- and acetylated alpha-tubulin were blocked with anti-mouse Fab fragments (20 μg/ml; Jackson Immunoresearch (cat # 715-007-003; lot # 114326)) as previously described [24]. Secondary antibodies were species-specific and were conjugated with fluorescent tags (1:400 (ICC/IHC); Jackson Immunoresearch). Stained sections and cells were coverslipped with Prolong Gold antifade media containing DAPI (Invitrogen).

### Immunostaining and quantification of ciliated cells

Stained sections and cells were examined and imaged using an Olympus IX81-DSU confocal microscope fitted with a 60x water objective. All images were captured as z-stacks (0.5 μm steps). The number of ciliated mCherry and mCherry/dnKif3a-positive cells and clones derived from single cell sorting was determined by counting the number of DAPI-labeled nuclei and aa-tubulin-positive cilia in each field/z-stack of randomly selected 4–6 microscopic fields per coverslip (3–5 coverslips/cell line and one coverslip/clone). The number of ciliated cells was expressed as a percentage of the total number of DAPI-labeled nuclei for each field. The mean background-corrected fluorescence intensity per pixel of Gli3C signal associated with aa-tubulin-positive cilia (n = 40 per group from 3 coverslips) was quantified using Image J software.

### Electron microscopy

As described previously [24], spheres of L0 or S3 cells grown in standard DMEM/F12 medium supplemented with hEGF and bFGF were pelleted at 600 G for 5 min, fixed, and resuspended in a mixture of 3% PFA and 2% glutaraldehyde for 1 h. The fixed spheres were washed in 0.1 M phosphate buffer (PB), post-fixed in 1% osmium tetroxide, dehydrated in ethanol, and embedded in resin. Ultrathin sections (70 nm) were collected onto grids, stained with uranyl acetate and lead citrate, and viewed using a Hitachi H-7600 transmission electron microscope at 80 kV. Images were captured with a Hitachi digital camera and proprietary software.

### RNA extraction, cDNA synthesis, and real-time quantitative PCR

RNA was extracted from cells 24 hours after vehicle or SHH treatment using the RNeasy Plus Universal Mini Kit (Qiagen cat # 73404) following manufacturer's instructions. On column DNase treatment was included in the extraction protocol (Qiagen cat # 79254) to remove residual genomic DNA carryover. cDNA synthesis was carried out using iScript Reverse Transcription Supermix for RT-qPCR kit (Bio-Rad cat #170-8840) per manufacturer's instructions, and input RNA for cDNA synthesis was 100 ng. RT reaction was carried out as follows: 5 min 25°C, 30 min 42°C, and 5 min 85°C. For real-time PCR, TaqMan primer probe assays (Life Technologies; all FAM-labeled) for human *GLI1*, *GLI2*, *GLI3*, *PTCH*, *SMO*, *SHH* (cat # 4453320), *SUFU* (cat # 4448892), and *β-actin* (cat # 4333762) were used, and amounts of each assay were used according to manufacturer's recommendations. KiCqStart® Probe qPCR ReadyMix (Sigma cat # KCQS04) was used for gene expression analysis per manufacturer's instructions. Real-time reaction was run on a Bio-Rad CFX96 Touch PCR system as follows: 1 cycle 95°C 30 sec, 45 cycles 95°C 15 sec 60°C 30 sec. Data was normalized to β-actin using the 2^−ΔΔCt^ methodology and the fold change between vehicle and SHH-treated samples was plotted.

### Intracranial xenografts

Male NOD/SCID mice (7-15 weeks old; Charles River) were used for our *in vivo* experiments. All procedures involving mice were performed according to NIH and institutional guidelines for animal care and handling. Mice were deeply anaesthetized using USP grade isoflurane (Halocarbon, North Augusta, SC). For each mouse, the scalp was reflected and a hole was drilled in the skull ∼2 mm lateral to Bregma. A Hamilton syringe was lowered 2.5 mm into the cortex, and 200,000 GBM cells, suspended in 2 μl of sterile culture medium, were slowly injected into the cortex over the course of 5 min. The syringe needle remained in place following delivery of the cells for an additional 5 min before it was removed. When they recovered from the surgery, the mice were returned to their home cages. Injected, tumor-bearing mice were monitored regularly and evaluated for tumor-related symptoms. Moribund animals were euthanized by CO_2_ inhalation and transcardially perfused with saline followed by 4% PFA. The brains were removed, post-fixed in 4% PFA overnight at 4°C, washed in PBS, and submerged in 30% sucrose. Once equilibrated, the brains were immersed in a 1:1 mixture of 30% sucrose:OCT overnight at 4°C. The brains were then frozen over liquid N_2_ in OCT and stored at −80°C until processing for histological analysis as described above.

### Data analyses

Statistical analyses were performed using GraphPad Prism 5.0 (GraphPad Software, La Jolla, CA). In all analyses, p-values less than 0.05 were considered significant. Comparisons of groups were done either using a one-way ANOVA or Student's t-test. Data that showed significant differences with ANOVA were further analyzed using Tukey's posthoc test. The Kaplan-Meier method and log rank test were used to analyze cell survival and create survival plots.

## SUPPLEMENTAL METHODS AND FIGURES


