# Supplementary material for: Disruption of KIF3A in patient-derived glioblastoma cells: effects on ciliogenesis, hedgehog sensitivity, and tumorigenesis
Source: Oncotarget. 2016 Jan 9;7(6):7029–43. doi: 10.18632/oncotarget.6854 (PMC4872766; doi:10.18632/oncotarget.6854)
Supplement: Supplementary file 1 [file oncotarget-07-7029-s001.pdf]

# SUPPLEMENTAL METHODS AND FIGURES

## Methyltetrazolium bromide (MTT), Trypan blue and caspase 3 assays

We performed two types of viability assays: MTT and Trypan blue assay. The MTT assay was performed as an indicator of cell viability as previously described [52]. Briefly, 10,000 cells were plated per well into 96 well cell culture plates and treated with vehicle, SHH (1 μg/ml) and/or cyclopamine with the same concentrations as above for cell counting experiments in 24 well plates. Bar graphs represent viable cell numbers through optical density measurements. Trypan blue was used to assess the number of dead cells following exposure to vehicle or SHH.

For the caspase 3 apoptosis assay, 250,000 cells were plated in T25 flasks and treated with vehicle or SHH (1 μg/ml) and fixed 5 days later for immunostaining. The primary antibody used was rabbit anti-cleaved caspase-3 (Asp175) (1:1000; Cell Signaling (cat # 9661S; lot # 42)). The secondary antibody was rabbit-specific and conjugated with fluorescent tag FITC (1:400; Jackson Immunoresearch). The percentage of caspase 3-positive cells was assessed using flow cytometry.

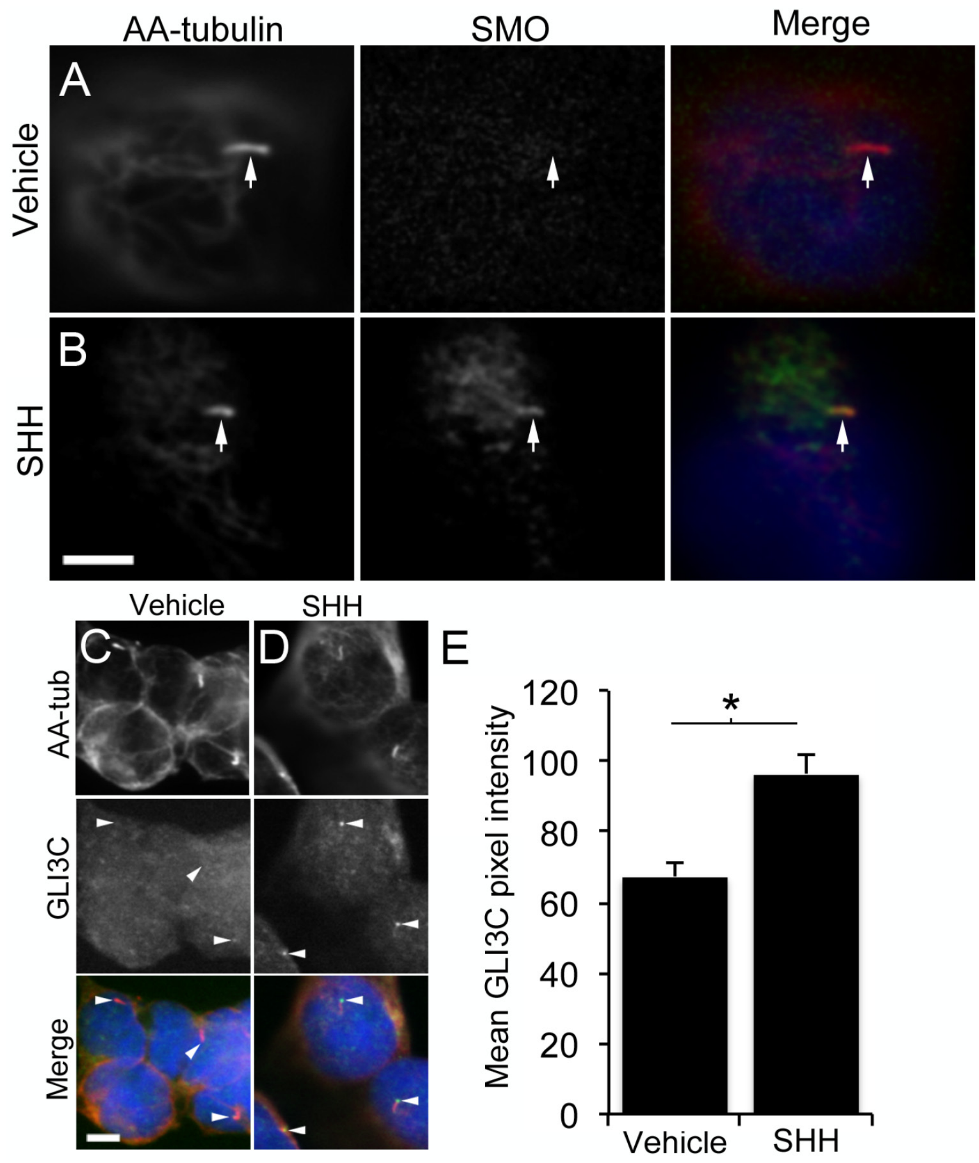


**Supplementary Figure S1: L0 cells engage components of the SHH signaling pathway.** (**A, B**) L0 cells were exposed to vehicle or SHH (1µg/ml). After 18 h, cells were fixed and stained for SMO and aa-tubulin. Unlike vehicle-treated cell cilia (arrow in **A**), we observed aa-tubulin-positive cilia (arrow in **B**) were also positive for SMO (green) after SHH treatment. (**C, D**) L0 mCherry-positive cells were grown on coverslips, fixed, and immunostained with antibodies against GLI3C (green) and acetylated alpha-tubulin (AA-tub; red) five days after exposure to vehicle or SHH (1 µg/ml). Confocal images show examples of ciliated cells in which GLI3 is enriched at the tip of aa-tubulin-positive cilia following SHH, compared to vehicle (compare arrowheads between C and D panels). (**E**) Quantification of the data from the staining shown in C and D is represented as mean GLI3C signal intensity per pixel (+/- SEM) of GLI3-positive cilia in vehicle or SHH-treated L0 cells (*$p<0.05$, Student's *t*-test). Scale bars for A and B = 10 µm; C and D = 10 µm.

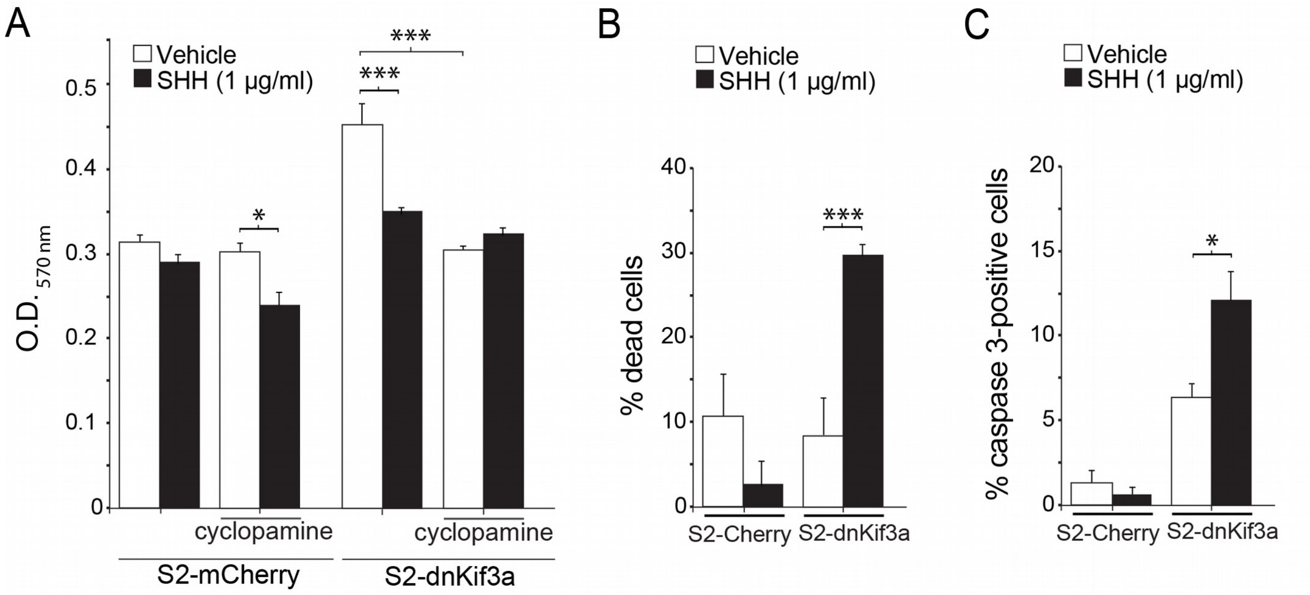


**Supplementary Figure S2: SHH exposure induces cell death/apoptosis in dnKif3a-expressing S2 cells.** (**A**) Cell viability represented as optical density (O.D.) measurement at 450 nm quantified by MTT assay ((+/- SEM) of mCherry versus mCherry and dnKif3a-expressing S2 cells following vehicle or SHH exposure, with or without cyclopamine pretreatment. (**B**) Percentage (+/- SEM) of dead mCherry versus mCherry and dnKif3a-expressing S2 cells following exposure to vehicle or SHH. Dead cells were labeled with trypan blue. (**C**) Percentage (+/- SEM) of active caspase 3-positive mCherry versus mCherry and dnKif3a-expressing S2 cells following exposure to vehicle or SHH. Labeled cells were quantified using flow cytometry. Groups were compared using a one-way ANOVA followed by Tukey's posthoc analysis. *$p$<0.05, ***$p$<0.005.

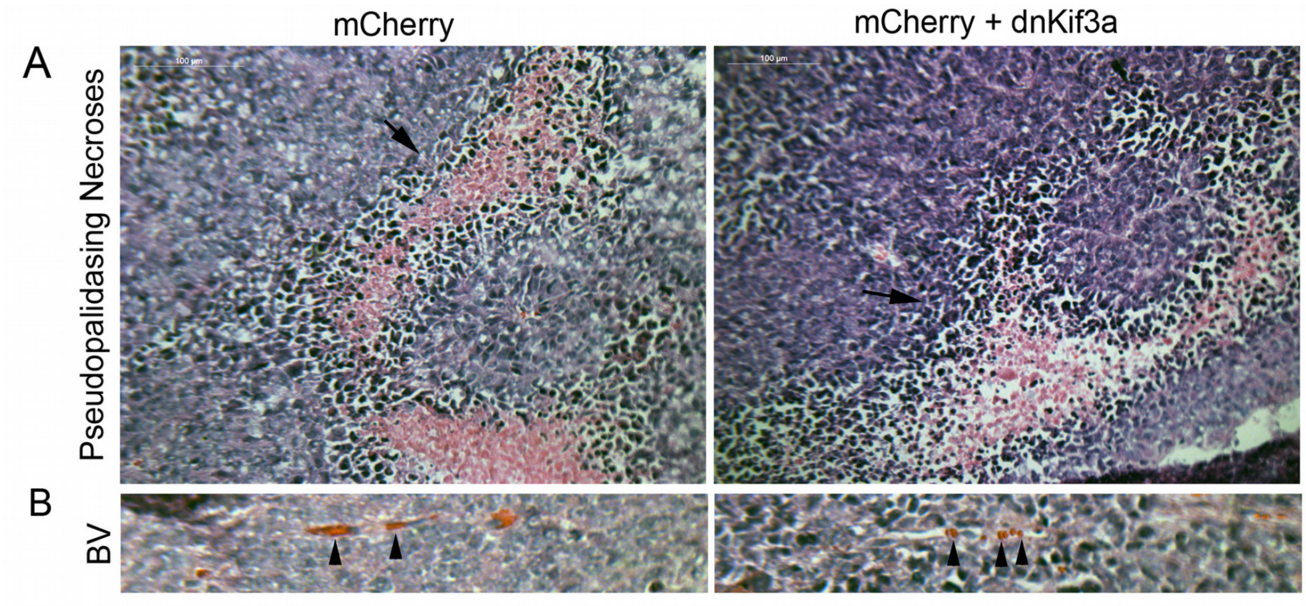


**Supplementary Figure S3: Pathological hallmarks of GBM persist in mCherry and mCherry and dnKif3a-expressing tumors.** (**A**) Hematoxylin and eosin staining of S3 mCherry (left) and mCherry and dnKif3a (right) tumors shows evidence of pseudopalisading necrosis formations (black arrows; upper panels). (**B**) Vascular proliferation was also evident in both tumors (lower panels). Arrowheads point to blood cells coursing through blood vessels (BV) in the tumor mass.

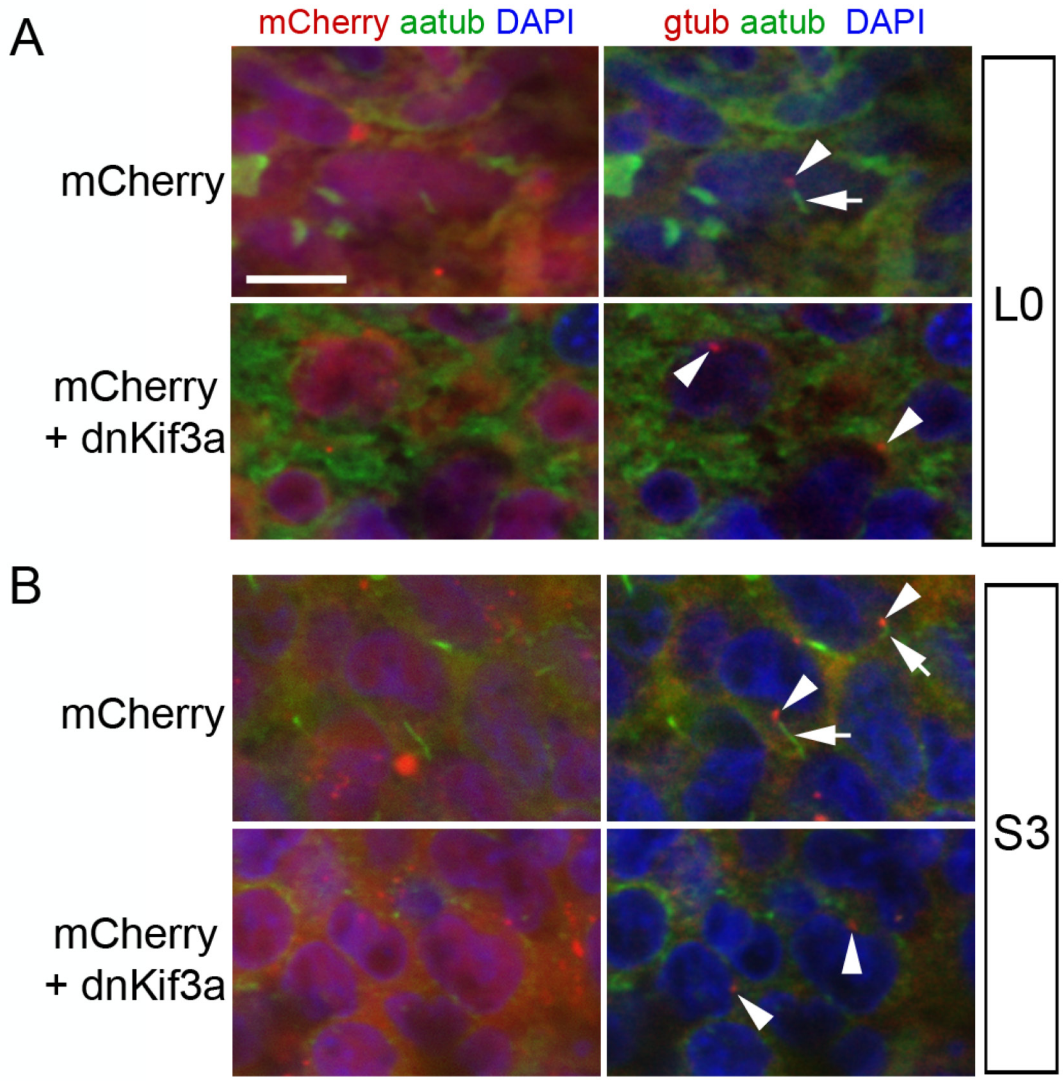


**Supplementary Figure S4: Acetylated tubulin-positive cilia are absent in dnKif3a-expressing tumors.** Sections through the core of mCherry-positive tumors (left panels) formed from L0 (**A**) and S3 (**B**) cell lines and immunostained for G-tubulin (gtub; arrowheads) and aa-tubulin (aatub; arrows). G-tubulin and aa-tubulin-positive cilia were detected in mCherry-positive control cells (upper panels in A and B), but only G-tubulin-positive basal bodies/centrioles, and no aa-tubulin-positive cilia, were observed in mCherry and dnKif3a-expressing cells (lower panels in A and B) in both cell lines. Scale bar for A and B = 10 µm.
